# P220: Epidemiology of nosocomial infections declared through the newly implemented computer-based infection declaration system (e-sin) in the hospitals of Paris region

**DOI:** 10.1186/2047-2994-2-S1-P220

**Published:** 2013-06-20

**Authors:** F Westphal, C Giroux, N Gaillard

**Affiliations:** 1 ARS (Regional Agency for Health), Paris, France

## Introduction

In order to centralize and simplify the nosocomial infection (NI) declaration process in France, the French Ministry of Health has developed a computer-based infection declaration program that has been implemented nationally in January 2012. This system allows each hospital physician running an Infection Control Unit to declare NI on a dedicated web site. Usefulness of declaring NI is evaluated by the hospital physician according to a number of predefined criteria that are reminded online in the declaration form (e.g., characteristics of the infecting microorganism, nature of the infectious site, context of acquisition of the NI, associated death, …). Additionally, declaration of colonization with multidrug-resistant bacteria is recommended.

## Objectives

We report the results of the first year of surveillance by this NI declaration program in the hospitals of Paris and Paris region.

## Methods

All infection declarations have been analyzed on a real-time basis.

## Results

In total, 345 infection declarations have been recorded, coming from 24% (94/397) of the total number of hospitals in the region. University hospitals accounted for 49% of the declarations. Surgery or acute care medicine departments and intensive care units provided 76% of all the declarations.

The nature or susceptibility profile of the microorganism justified 67% of all the declarations. Respectively, carbapenem-resistant *Enterobacteriaceae*, other carbapenem-resistant Gram-negative bacilli (i.e., *A. baumanii*, *P. aeruginosa*), and glycopeptide-resistant *E. faecium* accounted for 27%, 16%, and 13% of all the declarations. There was a linear increase (r=0.76, p<0.01) in the month-to-month number of declarations for multidrug-resistant bacteria over the year.

Overall, 773 patients had NI while 249 patients had colonization, primarily by multidrug-resistant bacteria. There were 69 patients with NI attributable to medical or surgical procedures, mostly digestive endoscopy and ophthalmologic surgery.

## Conclusion

Given the number of hospital beds in Paris region (75,478), underreporting of NI appears to be a significant concern during this one-year surveillance period. Irrespective of the infection declaration system, physician perception of the usefulness of declaring NI probably need to be stimulated.

## Disclosure of interest

None declared

